# Low limit of detection of the AlGaN/GaN-based sensor by the Kelvin connection detection technique

**DOI:** 10.1038/s41378-021-00278-7

**Published:** 2021-07-01

**Authors:** Hanyuan Zhang, Ying Gan, Shu Yang, Kuang Sheng, Ping Wang

**Affiliations:** 1 College of Electrical Engineering, Zhejiang University, 310027 Hangzhou, China; 2 Biosensor National Special Laboratory, Department of Biomedical Engineering, Zhejiang University, 310027 Hangzhou, China; 3 Hangzhou Global Scientific and Technological Innovation Center, Zhejiang University, 310027 Hangzhou, China

**Keywords:** Electrical and electronic engineering, Biosensors

## Abstract

The AlGaN/GaN-based sensor is a promising POCT (point-of-care-testing) device featuring miniaturization, low cost, and high sensitivity. BNP is an effective protein biomarker for the early diagnosis of HF (heart failure). In this work, a novel AlGaN/GaN device with the Kelvin connection structure and the corresponding detection technique was proposed. This technique can effectively suppress the background noise and improve the SNR (signal-to-noise ratio). A BNP detection experiment was carried out to verify the effectiveness of this technique. It is shown that compared with that of the traditional detection method, the LOD (limit of detection) was improved from 0.47 ng/mL to 1.29 pg/mL. The BNP detection experiment was also carried out with a traditional electrochemical Au-electrode sensor with the same surface functionalization steps. The AlGaN/GaN sensor showed a better LOD than the Au-electrode sensor. Moreover, the influence of AlGaN/GaN sensor package on background noise was investigated with the mechanism of the noise source revealed. Finally, based on the optimized package, the optimal SNR quiescent operating point of the AlGaN/GaN sensor was determined. By biasing the sensor at the optimal quiescent operating point and immobilizing the magnetic beads with anti-BNP on the gate of the AlGaN/GaN sensor, the LOD for BNP detection was further improved to 0.097 pg/mL.

## Introduction

Heart failure (HF) is a cardiovascular disease that is one of the leading causes of death worldwide. The detection of clinically relevant cardiac biomarkers is effective in allowing the early diagnosis of HF, reducing the complications and the risk of recurrence, and ultimately reducing the economic burden on the entire medical system. Brain natriuretic peptide (BNP) is one of the most promising clinical biomarkers of HF. Based on various reports^1,2^, the clinical cutoff level of BNP in chronic HF diseases is ~100 pg/mL. In addition, there are reports that BNP has predictive value in the diagnosis of anthracycline-induced cardiotoxicity (AIC)^3^, for which the diagnostic threshold is 107.9 pg/mL.

Currently, the clinical diagnostic systems used to detect protein biomarkers has drawbacks such as the need for relatively high volumes of blood samples, well-trained experts, large-scale equipment, and long processing times. A low cost, portable, and accurate POCT device is urgently needed for increased in-home monitoring of protein biomarkers for global epidemics such as HF.

The AlGaN/GaN FET sensor is a promising POCT device^4–6^. With the use of different functionalization processes, the AlGaN/GaN immunoFET^7^ can detect various disease biomarkers^4,5,8–10^. However, compared with other traditional electrochemical biosensors, such as Au electrodes, the manufacturing process for AlGaN/GaN FET devices is more complicated, GaN epitaxy is more expensive, and the device package design is more difficult, while the advantages of AlGaN/GaN sensors versus Au electrodes are not clear. In this work, by adopting the same surface functionalization method for the traditional Au-electrode electrochemical sensor and the AlGaN/GaN FET sensor (Device A in this work), the BNP detection performances of the two sensors were compared.

The two-dimensional electron gas (2DEG) structure formed by the AlGaN/GaN heterojunction can achieve both high carrier mobility (typically, ~2000 cm^2^/V ∙ s) and high electron density (typically, ~1 × 10^13^ cm^−2^)^11^; thus, the current sensitivity *S*_I_, which represents the ability of the AlGaN/GaN device to amplify the biosignal, is very high^12^. Therefore, methods to improve the sensitivity of AlGaN/GaN sensors have been extensively studied^11–19^. However, in practical applications, the background noise of the device may also be amplified, and the benefits of the high sensitivity will then be offset. As a result, it is very important to determine how to reduce the background noise of the device to improve the overall performance. To reduce the background noise and improve the SNR of the device, we proposed using a Kelvin connection device structure and the corresponding test method. The four-probe test method is an electrical measurement method used to accurately measure resistance while excluding the influence of the series resistance. The principle involves decoupling the current loop and the voltage measurement loop so that the current and voltage signals can be measured accurately and the resistance can be calculated accurately. The Kelvin connection technique was also used in traditional ISFET sensors^20^. However, the role of the Kelvin connection in ISFETs and AlGaN/GaN devices is different. In the ISFET device, the Kelvin connection technique is used to isolate the terminal voltage measurement loop from the current source loop. However, in the AlGaN/GaN sensor, it is used to suppress the background noise and improve the SNR. The difference in the Kelvin connection technique of the two devices originates from the differences in sensor structures and sensor characteristics. In the fabrication process of the CMOS-compatible ISFET, it is relatively difficult to produce a successful passivation layer to selectively expose the active sensing area and to protect the metal leads from the solution at the same time. Therefore, there are long source and drain regions that are highly doped with Si, and these are simplify the use of materials such as thick epoxy to cover the metal leads^21^. The highly doped drift regions provide excessive series resistance, which reduces the current sensitivity of the FET sensor^11^. Moreover, since the SiO_2_ gate oxide layer grown by the thermal oxidation process is easily penetrated by the ions in the solution, which causes the turn-on voltage shift and device failure, the poly gate is connected to the thick SiN_x_ layer acting as the passivation layer and the gate-sensitive membrane^21^. The SiN_x_ layer provides excessive series capacitance to the gate of the ISFET, and the current sensitivity is further reduced. Therefore, to compensate for the poor current sensitivity, source and drain follower read-out circuits are often used in sensor arrays to ensure that the reference electrode is grounded^22^. In the ISFET read-out circuit, both drain-to-source voltage (*V*_DS_) and drain-to-source current (*I*_DS_) are constant, and the change in the solution will cause a change in the gate voltage Δ*V*_G_ through the double layer on the solution/SiN_x_ interface. Δ*V*_G_ is equal to the output signal source voltage (*V*_S_) and is amplified by the off-chip amplifier. To accurately read the *V*_S_, the voltage readout circuit loop is decoupled with the current-flowing circuit loop based on the Kelvin connection technique. In the source and drain follower read-out circuit, the Δ*V*_G_ signal is not amplified, and the signal-to-noise ratio (SNR) is limited by the electronic noise of the read-out circuit instead of the ISFET device itself. In this work, the AlGaN/GaN sensor is biased with a constant *V*_DS_ voltage, and *I*_DS_ = Δ*V*_G_ × *g*_m_ is read out as the output signal (equivalent to *R*_DS_ = *V*_DS_/*I*_DS_ when *V*_DS_ is a constant). Δ*V*_G_ is amplified by *g*_m_, and the SNR is limited by both *g*_m_ and the noise of the AlGaN/GaN sensor itself. The reason for using the Kelvin connection technique in the AlGaN/GaN sensor is that according to our previous study^11^, the series resistance reduces the *g*_m_ of the sensor. The Kelvin connection technique can reduce the influence of the series resistance and improve *g*_m_. However, in the experiment, we unexpectedly found that the Kelvin connection technique also contributes a positive role in the noise performance of the device; it not only reduces the thermal white noise on the series resistance but also effectively reduces the overall 1/f mode background noise of the AlGaN/GaN sensor and improves the LOD.

Passivation is one of the key challenges in liquid sensors^7^. It is well known that the quality of the package will affect the device lifetime^23–27^ and the safe operating area (SOA)^28^. However, the influence of the package on the device background noise has not been discussed. PECVD-grown SiO_2_ and SiN_x_ are CMOS-compatible packaging materials often used as liquid sensors^17,18,29,30^. In our previous work, the PI/SiN_x_/SiO_2_ multilayer package was found to effectively suppress the leakage current *I*_G_ flowing between the metal and the reference electrode in the solution under different voltage stresses^31^. By comparing the correlation between *I*_G_ and the background noise for the PI/SiN_x_/SiO_2_ multilayer package and the traditional SiO_2_/SiN_x_ package, we found that the package material affects the device background noise by influencing the gate leakage current. The mechanism of the process was also illustrated by the experimental results.

The quiescent operating point of the AlGaN/GaN sensor is an important factor that influences the sensitivity^32^. However, the effect of the quiescent operating point on the SNR has not been investigated. In this work, the SNR of the AlGaN/GaN device with different quiescent operating points was determined. Another method to detect BNP was developed by immobilizing anti-BNP on the magnetic beads and adsorbing the magnetic beads on the surface of the AlGaN/GaN open gate area through a magnet^8^. The device (Device B in this work) was biased at the optimal quiescent operating point, and the LOD for BNP detection was further improved to 0.097 pg/mL.

## Results

### BNP detection: Au-electrode with EIS

**Fig. 1:**
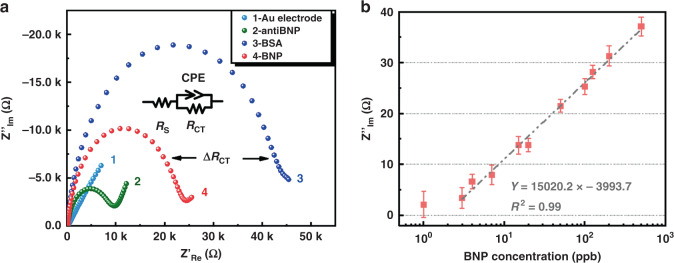
BNP detection results based on Au-electrode. **a** Change of *R*_CT_ in the EIS with the surface functionalization process. The inset is the circuit model used to simulate the *R*_CT_ value. Z′_Re_ is the real part, and Z′_Im_ is the imaginary part of the impedance in the EIS of the Au electrode. **b** Change in *R*_CT_ at different concentrations of BNP.

Figure 1a shows the change in the charge transfer resistance (*R*_CT_) of the Au electrode with the functionalization process in the Electrochemical Impedance Spectroscopy (EIS) test. The *R*_CT_ increased from 1 to 2 after the Au electrode was coated with BNP antibody (anti-BNP) and further increased from 2 to 3 after the binding sites were blocked by bovine albumin (BSA). When BNP and anti-BNP were specifically bound, *R*_CT_ decreased from 3 to 4, and the amount of *R*_CT_ decrease was Δ*R*_CT_. The inset shows the circuit model used to simulate the *R*_CT_ value in the EIS curve. *R*_S_ represents the series resistance, including the solution resistance and the series resistance in the circuit. *R*_CT_ is the charge transfer resistance, and constant phase element (CPE) is used to simulate the capacitance of the double layer and compensate for the nonhomogeneity in the system. Figure 1b shows the plot of Δ*R*_CT_ versus different BNP concentrations ranging from 1 ppb to 500 ppb. *R*_CT3_ is the charge transfer resistance of the Au electrode after BSA blocking, and the standard deviation is *σ*_*R*CT3_ = 849.4 Ω, which can be regarded as the background noise. Substituting *σ*_RCT3_ three times into Y of the linear fitting line of Δ*R*_CT_-BNP, the X value obtained is the LOD of BNP detection by the Au electrode. In this work, the LOD of the Au-electrode is 2.73 ppb.

The selectivity of the Au electrode treated with this functionalization method was determined, and the results are shown in Fig. S1 in the supplementary material.

### Fabrication of the AlGaN/GaN sensor

**Fig. 2:**
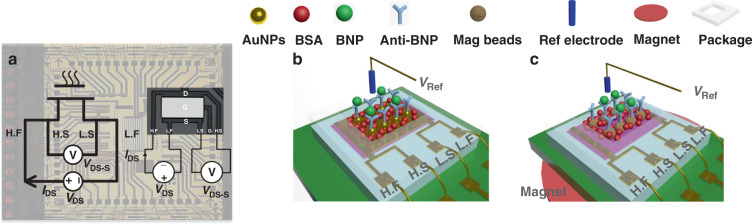
Schematic diagram and structure model diagrams of the Kelvin connection devices. **a** Diagram of the Kelvin connection device; high force (H. F) and high sense (H. S) are connected to the drain terminal, low force (L. F) and low sense (L. S) are connected to the source terminal. *V*_DS_ voltage is applied between the H. F and L. F to form the current loop. The voltage between the H. S and L. S is measured as *V*_DS−S_. The diagram on the left is the schematic diagram and that on the right is the OM (Optical microscope (OM) top view of the real device. **b** Device A. The open gate area of device A is deposited by the Au layer. AuNPs (Au nanoparticles) are electroplated on the Au gate, and the anti-BNP is immobilized on the AuNPs to selectively detect BNP. **c** Device B. The open gate area of Device B is bare AlGaN. Magnetic beads immobilized with anti-BNP are absorbed on the AlGaN surface with a permanent magnet on the back side of the chip.

Two types of AlGaN/GaN devices designed in this work, Device A and Device B, are shown in Fig. 2b, c. The fabrication and packaging process of Device B has been described^31^, and the only difference between Device A and Device B is that the open gate area was deposited with a layer of Au as the sensitive membrane. The PI/SiN_x_/SiO_2_ multilayer was used as the package in this work. There are four terminals for both Device A and Device B. High force (H. F) and high sense (H. S) terminals are connected to the drain, and low force (L. F) and low sense (L. S) terminals are connected to the source, and the circuit schematic and the actual device connection diagram are shown in Fig. 2a.

### BNP detection: AlGaN/GaN sensor (Device A) with Au gate

**Fig. 3:**
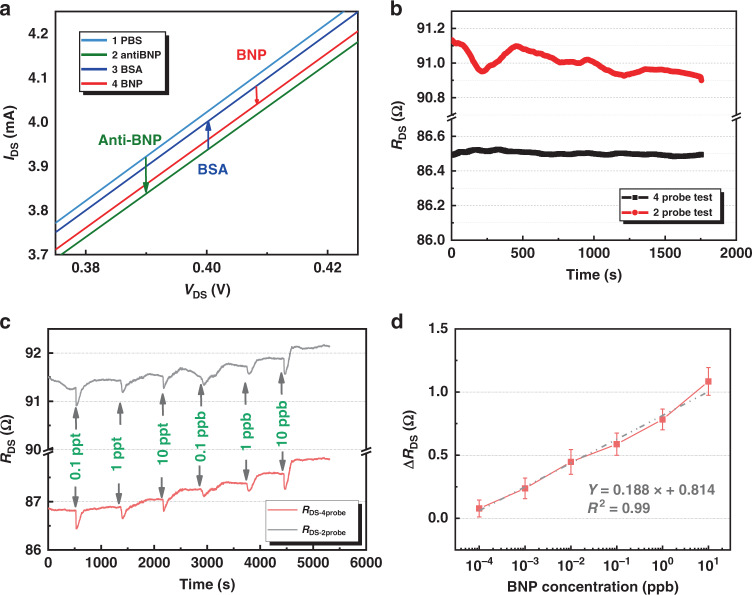
BNP detection results based on AlGaN/GaN sensor with Au gate membrane (Device A). **a** Change of *I*_DS_ output of Device A with the surface functionalization process. **b** *R*_DS_-t baseline comparison (*V*_DS_ = 0.4 V) between the Kelvin connection test and the traditional two-probe test. **c** *R*_DS-4probe_-t and *R*_DS-2probe_-t response to BNP with concentrations ranging from 0.1 ppt to 10 ppb using Device A as the transducer and anti-BNP immobilized on the Au gate. **d** Δ*R*_DS_ results of BNP detection.

Figure 3a shows the change in *I*_DS_ output with the functionalization process of Device A. The trend of *I*_DS_ change was similar to that of the Au electrode; after the Au gate was coated with anti-BNP, *I*_DS_ was reduced, and in other words, the channel resistance of the AlGaN/GaN device increased. Then, the Au gate was blocked by BSA, and the *I*_DS_ increased. Finally, after BNP and anti-BNP were specifically bonded, the *I*_DS_ decreased again.

Figure 3b shows the *R*_DS_ baseline measurement for the Kelvin connection technique and two-probe test method after the BSA blocking process. *V*_DS_ was fixed to be 0.4 V. It is shown that *R*_DS-4probe_ (drain-to-source resistance measured by Kelvin connection technique) was smaller than *R*_DS-2probe_ (drain-to-source resistance measured by two-probe method) by ~5 Ω in absolute value, and its variation was also smaller than that of *R*_DS-2probe_. The absolute value of *R*_DS-4probe_ was reduced because it eliminated the influence of the series resistance in both the measurement circuit and the thin film resistance of the wafer. The reduction in the resistance variation indicates that the series resistance can not simply be regarded as a constant resistance; it also introduced noise into BNP measurements, thereby affecting the LOD of the sensor. The standard deviation of the Kelvin connection technique *σ*_*R*DS-4probe_ was 0.09 Ω, while that of the two-probe method *σ*_*R*DS-2probe_ was 0.251 Ω.

Figure 3c shows the *R*_DS-4probe_ and *R*_DS-2probe_ responses to BNP at concentrations ranging from 0.1 ppt to 10 ppb, indicating that *R*_DS-4probe_ showed a more stable response than *R*_DS-2probe_. Figure 3d shows the plot of Δ*R*_DS_ (drain-to-source resistance change) versus different concentrations of BNP. Substituting 3*σ*_*R*DS-4probe_ into the linear fitting curve of Fig. 3d, the LOD of Device A using the Kelvin connection technique is 1.29 ppt. On the other hand, the LOD of the two-probe testing method is only 0.47 ppb due to the larger background noise.

The selectivity of Device A with this functionalization method was determined, and the results are shown in Fig. S2 in the supplementary material.

### Package and background noise

**Fig. 4:**
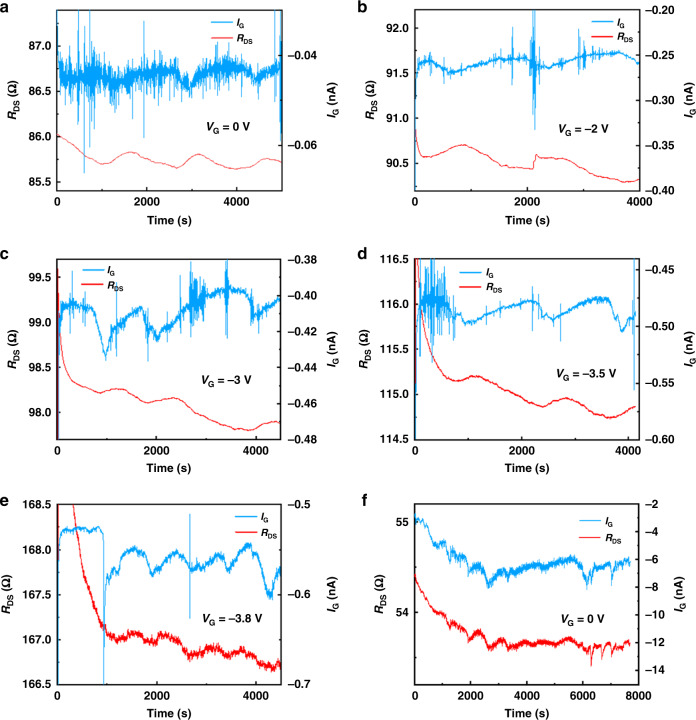
The experimental results of the gate leakage current *I*_G_ and *R*_DS_ background noise of the AlGaN/GaN sensors. **a**–**e** are the baseline tests of Device B with different *V*_G_ biases; the packaging material is PI/SiN_x_/SiO_2_. *R*_DS_ is the Kelvin connection test resuts and *I*_G_ is the gate leakage current through the reference electrode. *R*_DS_ and *I*_G_ were measured simultaneously with **a** *V*_G_ = 0 V, **b** *V*_G_ = − 2 V, **c** *V*_G_ = − 3 V, **d** *V*_G_ = − 3.5 V, **e** *V*_G_ = − 3.8 V. **f** Baseline test of Device B with *V*_G_ = 0 V; the packaging material is SiN_x_/SiO_2_.

Packaging materials need to ensure that the device can be safely operated in solution. An important indicator for characterizing packaging quality is the gate leakage current through the reference electrode of the device under different *V*_G_ bias voltages. This work showed that the gate leakage current affects the background noise in the *R*_DS_ baseline of the device. Therefore, optimizing the quality of the package not only directly expands the safe operating area of the device but also effectively reduces the background noise and enables the sensor to obtain a lower LOD. Figure 4a–e are the baseline test results of Device B with different *V*_G_ biases ranging from 0 V to −3.8 V. The packaging material was PI/SiN_x_/SiO_2_, which was demonstrated to effectively suppress the gate leakage current in prior work^31^. Figure 4f shows the baseline test of Device B with *V*_G_ = 0 V, but the packaging material was traditional SiN_x_/SiO_2_. The leakage *I*_G_ current was <10^−9^ A for the PI/SiN_x_/SiO_2_ package, while *I*_G_ was on the order of 10^−6^ A for the SiN_x_/SiO_2_ package. Moreover, it is obvious from Fig. 4f that *I*_G_ and *R*_DS_ showed a strong correlation.

### BNP detection: AlGaN/GaN sensor (Device B) with magnetic beads

The *I*_DS_ background noise *N*_IDS_ is obtained by calculating the standard deviation of the steady-state *I*_DS_ (500 ms/sample, sample time 2000 s) with *V*_DS_ = 0.4 V. The *I*_DS_ response Δ*I*_DS_ is obtained from the change in surface potential *ψ*_0_ multiplied by the transconductance *g*_m_. Thus, the SNR is defined as:

3 $${\mathrm{SNR}} = \frac{{\Delta I_{{\mathrm{DS}}}}}{{N_{{\mathrm{IDS}}}}} = \psi _0\frac{{g_{\mathrm{m}}(V_{\mathrm{G}})}}{{N_{{\mathrm{IDS}}}}}$$

**Fig. 5:**
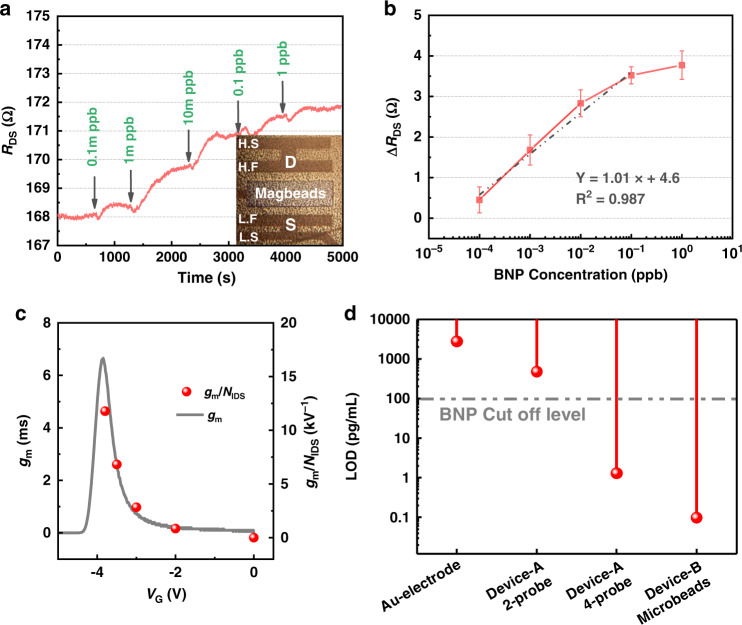
BNP detection results based on AlGaN/GaN sensor with magnetic beads (Device B). **a***R*_DS-4probe_-t response to BNP with concentrations ranging from 0.1 ppt to 1 ppb, using Device B as the transducer and anti-BNP immobilized on the magnetic beads. The inset is the optical microscope (OM) top view of the AlGaN/GaN device immobilized with magnetic beads. **b** Δ*R*_DS-4probe_ results of BNP detection with the Kelvin connection technique and *V*_G_ = −3.8 V. **c** Transconductance of Device B and its SNR versus different *V*_G_ biases of the reference electrode. **d** Comparison of the LOD values for the Au-electrode, Device A using the two-probe method, Device A using the Kelvin connection technique, and Device B biased at the optimal SNR quiescent working point (*V*_G_ = − 3.8 V), using the Kelvin connection technique.

Since *ψ*_0_ depends only on the interaction between the solution and the sensitive membrane, so *g*_m_/*N*_IDS_ represents the SNR of the device per voltage change in surface potential. Figure 5c shows the test results of *g*_m_ of Device B and its *g*_m_/*N*_IDS_ under different *V*_G_ biases. The SNR of Device B reached the optimal value at *V*_G_ = − 3.8 V.

The reason that Device A could not be operated with a negative *V*_G_ bias is that the SOA (safe operating area) of Device A is very small, so the Au metal on the gate region would be corroded^28^ at a negative *V*_G_ bias. To bias the device at the optimal SNR quiescent operating point, Device B (with a larger SOA) was used as the transducer. To immobilize the anti-BNP on the gate-sensitive area, anti-BNP was first immobilized on magnetic microbeads and then the microbeads were adsorbed on the open gate area of Device B with a magnet. When BNP specifically bonded with anti-BNP, it caused a potential change Δ*V*_G_ on the gate of Device B so that the channel resistance *R*_DS_ was read out as the output signal. The advantage of immobilizing the anti-BNP on the magnetic microbeads is that this method for detecting BNP can be used on Device B, which has a larger SOA. Moreover, the expensive AlGaN/GaN sensor can be easily refreshed and reused by removing the magnet and washing away the microbeads from the gate area. The BNP detection results are shown in Fig. 5a, b. The background noise of Device B at *V*_G_ = − 3.8 V was 0.18 Ω, and the calculated LOD was 0.097 ppt.

The selectivity of Device B with this functionalization method was determined, and the results are shown in Fig. S3 in the supplementary material.

## Discussion

**Fig. 6:**
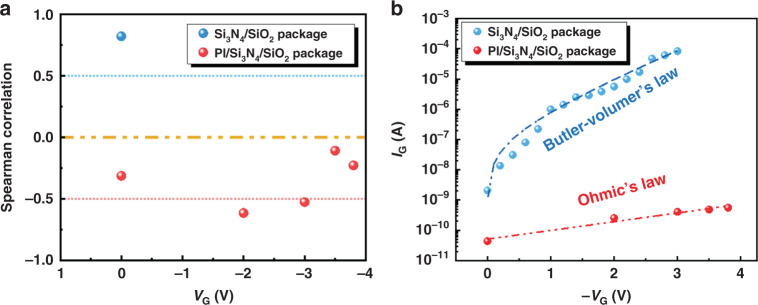
Influence of the packaging material to the background noies. **a** Spearman correlation of *I*_G_ and *R*_DS_ in Fig. 4. **b** Steady-state value of *I*_G_ with different *V*_G_ biases and different packages.

The Spearman correlation coefficients of the *I*_G_ and *R*_DS_ (Fig. 4) are compared in Fig. 6a. This result shows that for the PI/SiN_x_/SiO_2_ package, the gate leakage current *I*_G_ and the baseline noise *R*_DS_ presented a weak negative correlation between −0.62 and 0 with any quiescent working points of *V*_G_ from 0 V to −3.8 V. However, for the SiN_x_/SiO_2_ package, the gate leakage current *I*_G_ had a strong positive correlation of 0.83 with the baseline noise *R*_DS_ with *V*_G_ = 0 V.

To illustrate the mechanism causing this difference in the correlations of two types of package materials, the steady-state values of *I*_G_, excluding the capacitance effect under different *V*_G_ voltage stresses, are plotted in Fig. 6b. For the PI/SiN_x_/SiO_2_ package, the *I*_G_−*V*_G_ curve obeyed Ohm’s Law:

1 $$J = en\mu E = en\mu V/a$$

where *e* is the charge of an electron, *n* is the intrinsic carrier density, *µ* is the carrier mobility, *E* is the electric field of the current, *V* is the voltage, and *a* is the thickness of the material. This indicated that the high-resistance package layer completely blocked the contact between the metal and the solution, and most of the potential between the source/drain metals and the gate reference electrode fell on the package layer. The gate leakage current was dominated by the package layer featuring ohmic characteristics; this finding indicated that there were no external carriers injected into the package layer, and only the internal carriers were driven by the *V*_G_ voltage stress to form the leakage current *I*_G_. At this time, the leakage current level was not strong enough to cause a significant voltage fluctuation at the open gate area of the device; thus, the *R*_DS_ baseline was not strongly influenced by the *I*_G_, so the correlation between *I*_G_ and the background baseline *R*_DS_ was weak.

For the SiN_x_/SiO_2_ package, the *I*_G_−*V*_G_ curve obeyed the Butler–Volmer Law:

2 $$J = J_0\left[ {\exp \left( { - \frac{{\alpha nF\eta }}{{RT}}} \right) - \exp \left( {\frac{{\beta nF\eta }}{{RT}}} \right)} \right]$$

where *J*_0_ is the exchange current density, *R* is the ideal gas constant, *T* is the absolute temperature, *n* is the number of electrons participating in the reaction, *F* is the Faraday constant, *α* is the transfer coefficient of the oxidation reaction, *β* is the transfer coefficient of the reduction reaction, and *η* is the overpotential of the electrochemical reaction. This indicated that the SiN_x_/SiO_2_ package failed to thoroughly protect the source/drain metals from the solution. There were local areas of metal in direct contact with the solution. At this time, the *V*_G_ mainly fell on the interface between the metal and the solution, which led to the overpotential *η*. The electrochemical reaction was driven by *η*, and the rate of the electrochemical reaction became the dominant factor in gate leakage current *I*_G_ at this time. The electrochemical reaction rate was also related to the surface state at the interface, the concentrations of reactants, the convection velocity of the solution, and other factors, so there were large fluctuations in *I*_G_. Although only a small part of the metal was in contact with the solution, the *I*_G_ was much larger with this packaging system than that in the PI/SiN_x_/SiO_2_ package. This fluctuating leakage current *I*_G_ flowed through the solution resistance and formed a fluctuating voltage drop on the open gate area of the device, which was further reflected in the device baseline as the *R*_DS_ fluctuation, in other words, as the background noise. When *I*_G_ decreased (the absolute value increased), the open gate voltage of the device increased, so the channel resistance *R*_DS_ decreased. As a result, we found that there was a strong positive correlation between *I*_G_ and *R*_DS_ for the SiN_x_/SiO_2_ package.

Consequently, the quality of the packaging material affects the leakage current on the reference electrode and then affects the background noise of the device. If the packaging material is perfect and the leakage current on the reference electrode obeys Ohm’s law, then *I*_G_ and *R*_DS_ show weak correlations, and other effects dominate the background noise. On the other hand, if the packaging material is poor, a local area of the metal electrode is in contact with the solution, and *I*_G_−*V*_G_ obeys the Butler–Volmer Law. Then, *I*_G_ and *R*_DS_ show a strong positive correlation, and the leakage current dominates the background noise. Therefore, high-quality packaging not only increases the lifetime of the device and enlarges the safe operating area but also reduces the background noise of the device, thereby increasing its LOD.

In summary, the LODs for BNP detection by the Au electrode, Device A using the two-probe method, Device A using the Kelvin connection technique, and Device B using magnetic beads as the sensitive membrane are compared in Fig. 5d. Compared with the Au electrode, the AlGaN/GaN FET device, which was made with exactly the same functionalization process, improved the BNP LOD from 2.73 ppb to 0.47 ppb. The Kelvin connection technique designed in this work was proven to effectively reduce the background noise and increase the LOD to 1.29 ppt, which was lower than the cutoff concentration for HF disease. This method can be used not only in the AlGaN/GaN device but also in any other kind of FET sensor. The quality of the package was very important to the AlGaN/GaN sensor, since it can affect the magnitude of the leakage current to the reference electrode and thereby influence background noise. The optimal SNR was also shown in this work. It was found that as long as the package effectively protected the metal from undergoing electrochemically reactions with the solution, the optimal SNR quiescent operating point roughly coincided with the peak *g*_m_ point. Biasing the device at the optimal SNR working point led to an extremely low LOD of 0.097 ppt. Table 1 compares the LODs exhibited by various immunosensors for various proteins. Table 1 also shows comparisons of the LOD for BNP detection in this work (Device B) with LODs of other sensors used to detect BNP, AlGaN/GaN sensors used to detect other proteins with a similar method (antigen antibody specific binding) and other two-dimensional material devices and nanodevices. Compared with the electrochemical sensors described in references^33,34^, the LOD is this work is more than 40 times smaller. Compared with other AlGaN/GaN immunosensors in references^35–40^, the LOD is more than 3 orders of magnitude lower. Compared with the graphene sensor and the silicon nanowire in references^36,37^, the LOD is approximately 4 orders of magnitude lower.

**Table 1:** Comparison of LODs for detection of different proteins by different transducer platforms

Transducer	Bioprobe	Target molecule	LOD	Refs.
AlGaN/GaN	Anti-BNP on microbeads	BNP	97 fg/mL	This work
Screen-printed carbon electrodes	Peroxidase-labeled BNP antibodies on gold nanoparticles	BNP	4 pg/mL	^33^
Silver disk electrode	Acetylcholinesterase-labeled anti-BNP antibodies	BNP	10 ng/mL	^34^
AlGaN/GaN HEMT	NT-proBNP specific aptamer	NT-proBNP	0.22 ng/mL	^35^
AlGaN/GaN HEMT	FHC antibody	Protein–peptide	56.7 ng/mL	^38^
AlGaN/GaN HEMT	Botulinum antibody	Botulinum toxin	1 ng/mL	^39^
AlGaN/GaN HEMT	Anti-NT-proBNP	NT-proBNP	181 pg/mL	^40^
Graphene	Human anti-EGP	EGP	1 ng/mL	^36^
Silicon nanowire	Anti-APOA1	hAPOA1	1 ng/mL	^37^

## Materials and methods

### Surface functionalization and BNP detection

For the Au electrode, the first step was to perform surface activation in a 0.1 M H_2_SO_4_ solution with a cyclic voltammetry (CV) sweep. Then, AuNPs (Au nanoparticles) were electroplated on the Au electrode with a constant *V* = −0.2 V sweep for 200 s in a 0.25-mM chloroauric acid solution. Subsequently, the Au electrode was incubated in 10 µg/mL anti-BNP in a 37 °C incubator for 3 h. The Au electrode was rinsed with PBST solution (PBS:Tween-20 = 1000:1) to remove the excess anti-BNP. BSA solution (5 mg/mL in PBS) was used to block sites on the Au electrode for 30 min in a 37 °C incubator and the electrode was again cleaned with PBST. Finally, the Au electrode was incubated in solutions with different concentrations of BNP at 37 °C for 1.5 h. EIS (electrochemical impedance spectroscopy) was measured in a mixed solution containing 2 mM potassium ferricyanide, 2 mM potassium ferrocyanide and 0.1 M KCl after each functionalization step.

For Device A, the functionalization steps before BNP binding were exactly the same as those of the Au electrode. The Ag/AgCl reference electrode was biased at 0 V. A constant voltage of 0.4 V, *V*_DS_, was applied between H. F and L. F, and the drain-to-source current *I*_DS_ was measured with a Keithley (USA) 2602B SMUA. The voltage *V*_DS-S_ between H. S and L. S was measured with SMUB. *I*_DS_ and *V*_DS-S_ were sampled simultaneously every 500 ms. Traditionally, *I*_DS_ is used as the output signal to monitor the change in BNP concentration in the solution. For the Kelvin connection technique, *R*_DS-4probe_ = *V*_DS-S_/*I*_DS_ was monitored as the output signal. Since *V*_DS_ is a constant, *I*_DS_ is equivalent to *R*_DS-2probe_ = *V*_DS_/*I*_DS_. Theoretically, *R*_DS-4probe_ − *R*_DS-2probe_ should be a constant *R*_S_, which is the series resistance in the circuit. Different concentrations of BNP (diluted in 0.01×PBS solution) were spiked onto the FET gate area, and the responses of the *R*_DS-4 probe_ and *R*_DS-2 probe_ were monitored.

For Device B, 10 µL magnetic beads (30 mg/mL) were mixed with 10 µL anti-BNP (0.5 mg/mL) and incubated for 6 h at 37 °C. Then, MBs were collected with a permanent magnet, and the supernatant was removed. The magnetic beads were washed 3 times with PBS, and then the binding sites were blocked by immersion in 1 mg/mL BSA solution for 0.5 h. Finally, magnetic beads with a concentration of 5 mg/mL were added to the surface of Device B and held with an N52 permanent magnet on the back side of the chip. The reason that we did not use the electromagnet was to avoid heat dissipation by the electromagnet, which may influence the temperature of the sensor. In the baseline test with different quiescent working points, the reference electrode was biased from 0 V to −3.8 V (0 V, −2 V, −3 V, −3.5 V, −3.8 V), and the *R*_DS-4probe_ and the gate leakage current *I*_G_ were both monitored versus time. In the BNP measurement experiment, the change in *R*_DS-4probe_ was monitored when different concentrations of BNP were spiked on the open gate area.

All experiments for the 3 types of transducers were repeated at least 3 times to ensure that the results were consistent.

### Reagents and materials

The Au-electrode CHI101 was purchased from CH Instruments Ins. Anti-BNP (ab20984) and BNP (ab87200) were purchased from Abcam. Magnetic beads (Dynabeads™ M-280 Tosylactivated) were purchased from Thermo Fisher Scientific.

## Supplementary information

Supplementary materials
