# Correction to: Low limit of detection of the AlGaN/GaN-based sensor by the Kelvin connection detection technique

**DOI:** 10.1038/s41378-021-00283-w

**Published:** 2021-08-02

**Authors:** Hanyuan Zhang, Ying Gan, Shu Yang, Kuang Sheng, Ping Wang

**Affiliations:** 1 College of Electrical Engineering, Zhejiang University, 310027 Hangzhou, China; 2 Biosensor National Special Laboratory, Department of Biomedical Engineering, Zhejiang University, 310027 Hangzhou, China; 3 Hangzhou Global Scientific and Technological Innovation Center, Zhejiang University, 310027 Hangzhou, China

**Keywords:** Electrical and electronic engineering, Biosensors

Correction to: *Microsystems & Nanoengineering*

10.1038/s41378-021-00278-7 published online 1 July 2021

Following publication of the original article^1^, it was noted that affiliation 3 was omitted. Affiliation 3 has been listed below.

Hangzhou Global Scientific and Technological Innovation Center, Zhejiang University, 310027 Hangzhou, China.

The original article [1] has been updated.
