# Supplementary material for: Low limit of detection of the AlGaN/GaN-based sensor by the Kelvin connection detection technique
Source: Microsyst Nanoeng. 2021 Jul 1;7:51. doi: 10.1038/s41378-021-00278-7 (PMC8433396; doi:10.1038/s41378-021-00278-7)
Supplement: Supplementary file 1 — Supplementary materials [file 41378_2021_278_MOESM1_ESM.docx]

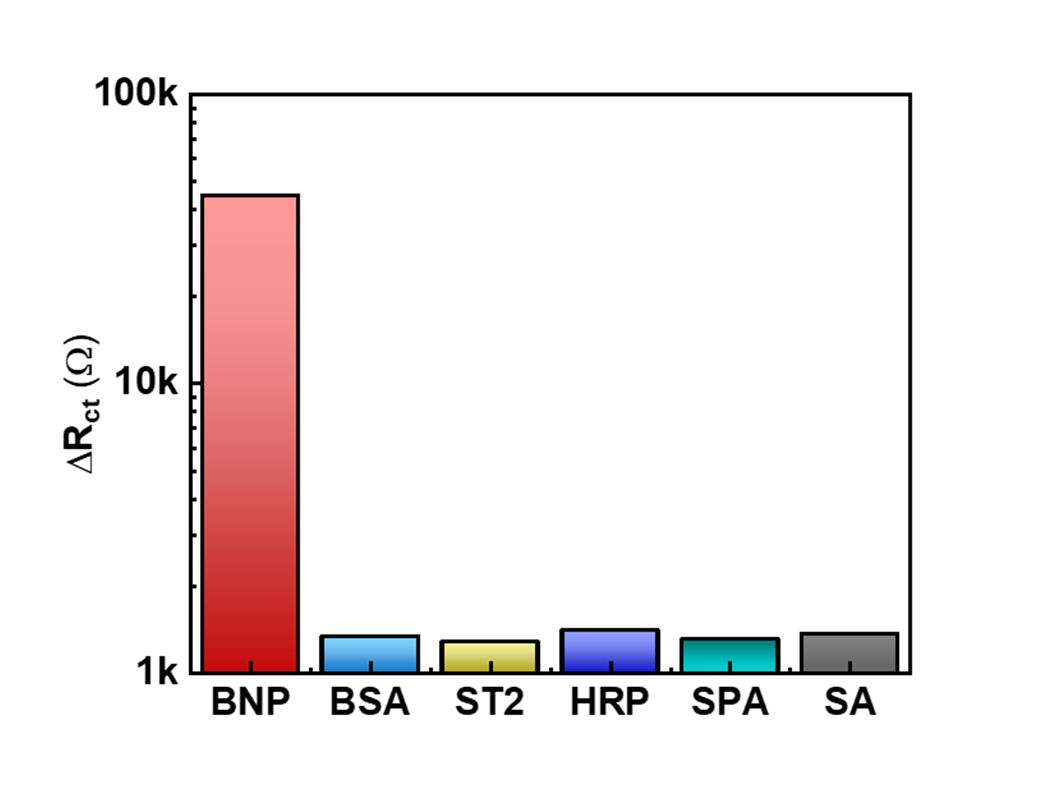


Fig. S1. The BNP and other proteins with concentrations of 1000 ppb are used to test the selectivity of the Au electrode.


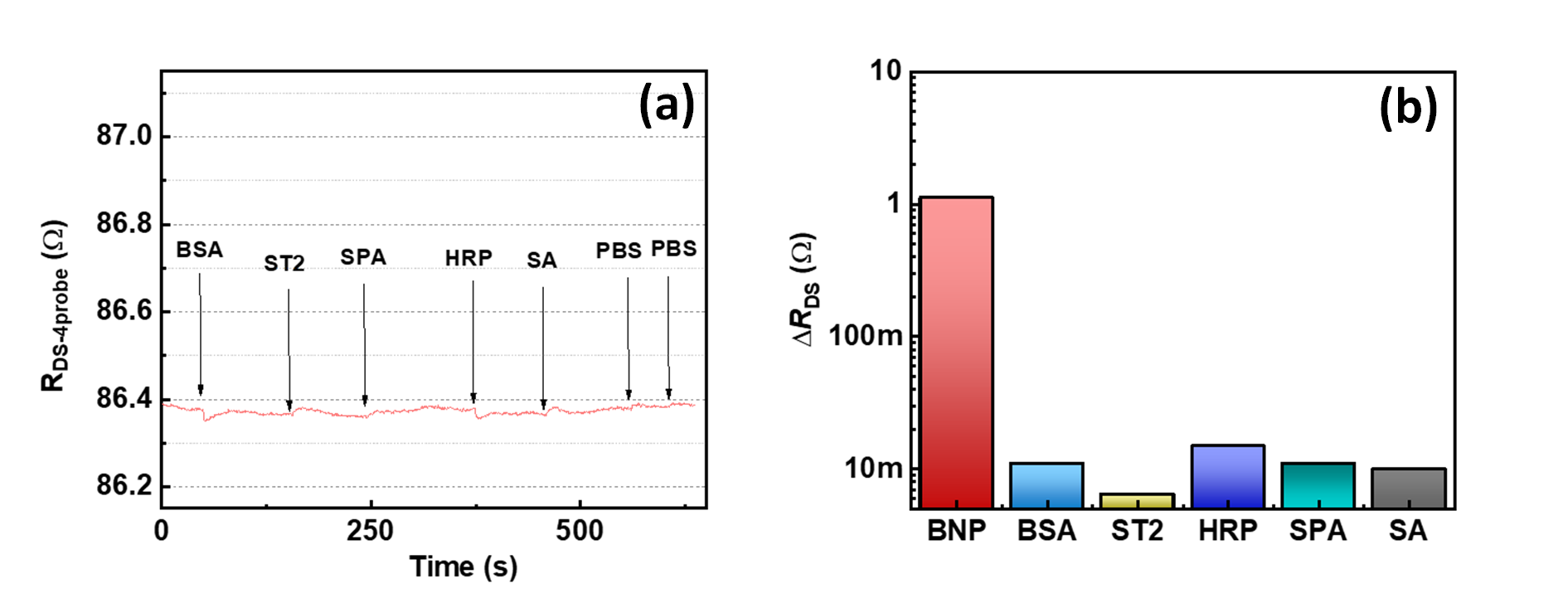


Fig. S2. The BNP and other proteins with concentrations of 10ppb are used to test the selectivity of the AlGaN/GaN sensor with Au gate.


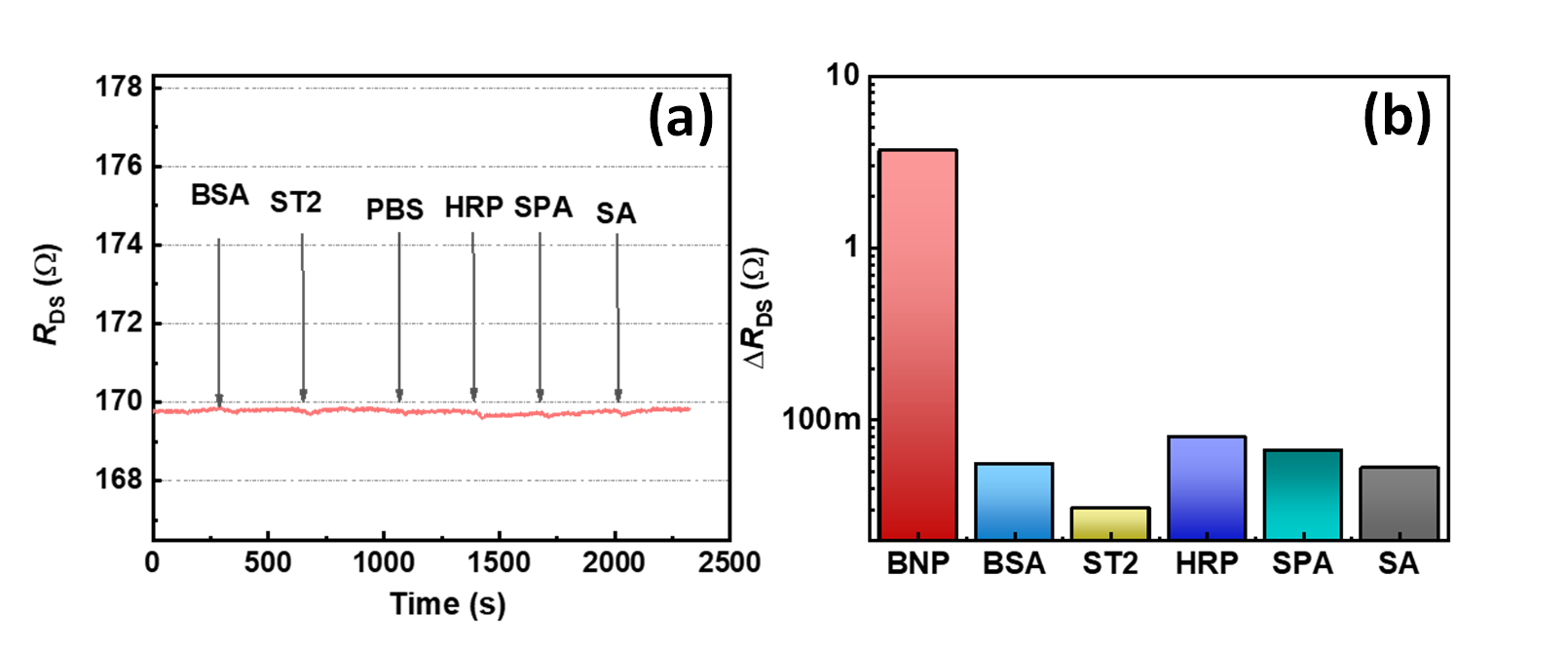


Fig. S3. The BNP and other proteins with concentrations of 1ppb are used to test the selectivity of the AlGaN/GaN sensor with magnetic beads immobilized with anti-BNP.
